# Clinical Utility of S.T.O.N.E, Guy's Scoring System, and Renal Stone Complexity Scoring in Predicting Outcome of Single-Tract Percutaneous Nephrolithotomy

**DOI:** 10.7759/cureus.50983

**Published:** 2023-12-23

**Authors:** Kausar Illahi Bux, Raja Rizwan Ahmed, Faryal Farooq, Nikhil Reddy Daggula, Jawad Mahmood, Umaima Wasim, Sajana Kumari, Muneeb Jan, Faheemullah Khan, Usha Kumari

**Affiliations:** 1 Radiology, The Kidney Center, Karachi, PAK; 2 Radiology, Jinnah Postgraduate Medical Center, Karachi, PAK; 3 Urology, The Kidney Centre, Karachi, PAK; 4 Diagnostic Radiology, Jinnah Post Graduate Medical Centre, Karachi, PAK; 5 Internal Medicine, Kakatiya Medical College, Warangal, IND; 6 Gastroenterology and Hepatology, Hayatabad Medical Complex Peshawar, Peshawar, PAK; 7 Medicine, Ziauddin University, Karachi, PAK; 8 Medicine, Dow University of Health Sciences, Karachi, PAK; 9 Internal Medicine Department, Khyber Teaching Hospital, Peshawar, PAK; 10 Medicine, Rehman Medical Institute, Peshawar, PAK; 11 Cardiology, Lady Reading Hospital, Peshawar, PAK; 12 Radiology, Aga Khan University Hospital, Karachi, PAK

**Keywords:** modified clavien grading, renal stone surgery, stone score, kidney stone disease, seoul national university renal stone complexity, renal stone disease, stone-free rate, percutaneous nephrolithotomy (pcnl), guy's score, s.t.o.n.e. scoring

## Abstract

Background: Several imaging-based scores have been developed to predict postoperative stone-free state (SFS) and complications. This study aimed to assess the accuracy of the S.T.O.N.E., Guy Scoring System (GSS), and Seoul National University Renal Stone Complexity (S-ReSCS) scores in predicting the outcomes of single-tract percutaneous nephrolithotomy (ST-PCNL). This scoring system holds paramount importance for low-income and low-middle-income countries (LMICs), as it is inexpensive and cost-effective for the healthcare system.

Methodology: This retrospective study was carried out with 147 participants. Based on the preoperative computerized tomographic (CT) scan, each patient's S.T.O.N.E. score, GSS, and S-ReSCS were recorded. The modified Clavien grading system was used to document intra- and postoperative complications.

Results: The mean age of the sample population was 45 years. SFS was achieved in 110 (74.8%) patients. The number of calyces involved (p = 0.008), S.T.O.N.E. scoring (p = 0.001), GSS (p = 0.008), and S-ReSCS (0.001) correlated well with the SFS. Forty-nine (33.33%) patients developed complications. The most common complications fell within Clavien grade II. No statistical significance was noted between the S.T.O.N.E. score, GSS, and S-ReSCS with the modified Clavien grading system.

Conclusion: The S.T.O.N.E. scoring, GSS, and S-ReSCS have a high predictive value for achieving SFS in ST-PCNL. In addition, findings from LMICs are comparable with those from the rest of the world.

## Introduction

Percutaneous nephrolithotomy (PCNL) is the preferred treatment for kidney stones. PCNL has evolved dramatically since its inception. Smaller stones were previously treated with extracorporeal shockwave lithotripsy (ESWL), while only larger stones were treated with PCNL. With the advancement in the field of optics, even smaller calculi are removed via PCNL with favorable outcomes [1]. Stones greater than 20 mm, staghorn, and partial staghorn calculi are better treated with PCNL. PCNL has demonstrated a stone-free rate of 85-93% [1]. The American Urological Association (AUA) guidelines propose PCNL as the modality of choice for staghorn stones, especially larger stones that occupy the inferior pole are best treated with PCNL. PCNL has higher stone clearance rates than ESWL, with comparable failure and complication rates [1,2].

To predict a stone-free state (SFS) after PCNL, multiple imaging-based preoperative scores have been introduced. The four commonly used scoring systems are S.T.O.N.E., GSS, Seoul National University Renal Stone Complexity (S-ReSCS), and Clinical Research Office of the Endourological Society (CROES). There are multiple benefits to having a standardized scoring system to predict the SFS [3-5]. PCNL results are skill dependent. This is the first study of its type being conducted on the Pakistani population. Therefore, it reflects the skills and efficacy of Pakistani surgeons in achieving the SFS following PCNL. We attempt to evaluate the reliability of three of the scores commonly used, i.e., S.T.O.N.E., GSS, and S-ReSCS, in predicting the SFS after a PCNL.

## Materials and methods

The study was carried out at the Kidney Centre, Karachi, Pakistan. Patients’ records were reviewed in retrospect for demographics, clinical, and laboratory parameters. Patients between January 2019 and June 2020 who had a computerized tomography of the kidneys, ureters, and bladder (CT KUB ) performed and underwent a single-tract PCNL (ST-PCNL) for renal stones were included. Patients under the age of 18 and those with bleeding diathesis were excluded. The sample size was determined using the World Health Organization's (WHO) sample size calculator, with a 10% assumed prevalence of kidney stones, a 95% confidence interval, and a 5% margin of error.

S.T.O.N.E., GSS, and S-ReSCS scores were documented for every patient based on a preoperative CT scan. The intraoperative and postoperative complications were recorded using the modified Clavien grading system. A follow-up X-ray or ultrasound of the KUB was performed to determine whether or not SFS was achieved. SFS was characterized by the lack of residual fragments measuring ≥4 mm. Statistical Package for the Social Sciences, version 20, was used for statistical analysis. For categorical variables, frequency and percentages were calculated, and the mean (standard deviation) was calculated for numerical variables. To establish significance, the independent t-test, Chi-square test, or Fischer exact test were used. Non-normally distributed data was presented as median with interquartile range (IQR), and the Mann-Whitney U test was used to compare them. The exemption was granted by the corresponding institute's Ethical Review Committee. The abstract of the study has been approved as an electronic poster (EPOS) at the European Congress of Radiology 2022.

## Results

The study included 147 patients, the majority of whom were males (70.7%). The average age of the participants was 45 years, and their average weight was 68.5 kg. 69 (46.7%) patients had stones on their left side, 68 (46.3%) had stones on their right side, and 10 (6.7%) had stones on both sides. The average size of the stone causing obstruction was 392.28 mm2, and in most of the patients (54.4%), it involved 1-2 calyces. Twenty-four (16.3%) patients had no obstruction, while 61 (41.5%) presented with mild, 53 (36.1%) with moderate, and nine (6.1%) presented with severe obstruction.

The most common complications fell within Clavien grade II. SFS was achieved in the majority of the patients (74.8%). SFS correlated well with the number of calyces involved (S.T.O.N.E., GSS, and S-ReSCS). No statistical significance was noted between S.T.O.N.E. scoring, GSS, and S-ReSCS with the modified Clavien grading system. Mean serum potassium levels decreased from 6.43 mg/dL (preoperative) to 4.11 mg/dL (on postoperative days 1 and 2). Following surgery, 27% of patients experienced a fever, 17% reported urinary leakage, 15% needed blood transfusions, and 5.4% experienced collections near the kidney. None of the patients experienced postoperative urinary retention. Table 1 illustrates the remaining details.

**Table 1:** Association between descriptive variables and stone-free state *p value < 0.05

Parameters	Number (%)	Mean ± S.D.
Gender		
Male	79 (71.8)	
Female	31 (28.2)	
Age (years)		46 ± 15
History of previous surgery	43 (39.1)	
Side of stone		
Right-sided stone	53 (48.2)	
Left-sided stone	50 (45.5)	
Both sided stone	7 (6.4)	
Weight (Kg)		68 ± 18
Guy's stone score*		
Grade I	33 (30.0)	
Grade II	47 (42.7)	
Grade III	19 (17.3)	
Grade IV	11 (10.0)	
S.T.O.N.E score*		2 (2)
Stone size (mm^2^)		388 ± 284
Tract length (mm^2^)		6.49 ± 1.39
Obstruction		
None	18 (16.4)	
Mild	50 (45.5)	
Moderate	36 (32.7)	
Severe	6 (5.5)	
Number of calyces involved*		
1-2	68 (61.8)	
3	30 (27.3)	
Staghorn	12 (10.9)	
Essence (Hounsfield Units)		959 ± 375
Renal stone complexity score (S-ReSCS)*		6.85 ± 1.36
Operation duration (hours)		2.78 ± 0.82
Modified Clavien grading system		
1	17 (15.5)	
2	14 (12.7)	
3a	3 (2.7)	

The area under the curve (AUC) for Guy’s score was 0.644, the S-ReSC score was 0.684, and the S.T.O.N.E. score had an asymptotic significance of <0.05 for each. The three scores had similar predictive accuracy for SFS, as illustrated in Table 2.

**Table 2:** Area Under the Curve (Asymptotic Confidence Interval 95%) S-ReSCS: Seoul National University Renal Stone Complexity

Test Result Variable(s)	Area	Std Error	P-value	Lower Bound	Upper Bound
S.T.O.N.E score	0.680	0.047	0.001	0.587	0.773
Guy’s score	0.644	0.047	0.009	0.551	0.737
S-ReSCS score	0.684	0.048	0.001	0.589	0.778

## Discussion

PCNL is preferred for kidney stones because of its minimally invasive nature. The procedure has almost replaced open surgery procedures for stone removal due to its decreased postoperative complications, short procedure, lesser cost, and decreased morbidity [6]. Despite the favorable outcomes of PCNL in most cases, there is still a risk of complexity for patients undergoing PCNL. Multiple scores and tools are introduced to preoperatively predict the complexity of the PCNL procedure, SFS, and associated complications. Radiology plays a central role in this aspect. To this date, there is no universally acceptable scoring system.

The SFS was achieved in 74.8% of the population. This result is comparable to what has been reported by other studies [4,7-9]. The reason for the slightly lower percentage of patients with SFS might be the liberal inclusion criteria we used. S.T.O.N.E. scoring showed a significant association with SFS after ST-PCNL (p = 0.001). The score combines five parameters, including the size of the stone, length of the tract, presence or absence of obstruction, number of calyces involved, and radiodensity of the stone. The number of involved calyces showed a significant association with SFS (p = 0.008). Many other studies have also reported that the S.T.O.N.E. score is significantly associated with SFS [4,10,11].

GSS was developed by Thomas et al. to classify stones preoperatively and predict procedural outcomes. The score is comprised of four grades: the higher the grade, the more complex the stone is. In our case, lower GSS was more likely to result in SFS (p = 0.008). Multiple other studies investigating the reliability of the score have also shown a good correlation between GSS and SFS [3,4,12-14]. S-ReSCS is a score more focused on the structural complexity of the stones. The score represents the number of sites involved, irrespective of stone size and number. In our study, S-ReSCS correlated well with SFS after ST-PCNL (p = 0.001). A similar correlation between the S-ReSCS and SFS has also been reported previously [5,15,16].

The modified Clavien grading system was used for evaluating perioperative and postoperative complications after PCNL procedures. The system classifies the postoperative complications into five grades: Grades 1 through 5. Grade 1 represents any deviation from the normal postoperative course without the need for intervention, and grade 5 represents the death of the patient [17]. The most common complications in our study fell within grade 2 of the modified Clavien grading system. Fever was the most common complication noted. Previous studies documenting post-PCNL complications have reported similar findings [4,18,19].

Due to the study's retrospective design, it has its limitations. A relatively small sample size is also one of the limitations. Since this was a single-center retrospective study, a larger, multi-center study may be necessary to validate the study results.

## Conclusions

The scores for S.T.O.N.E., GSS, and S-ReSCS are highly predictive of SFS in ST-PCNL. These scores are an easy and valuable tool to prognosticate PCNL outcomes and hence should be incorporated into routine preoperative use. Outcomes from LMICs are comparable to those of the rest of the world. The use of these tools prior to surgery may also assist practitioners in providing counseling to their patients and determining the need for redo surgery.
